# DTGHAT: multi-molecule heterogeneous graph transformer based on multi-molecule graph for drug-target identification

**DOI:** 10.3389/fphar.2025.1596216

**Published:** 2025-04-28

**Authors:** Xinchen Jiang, Lu Wen, Wenshui Li, Deng Que, Lu Ming

**Affiliations:** ^1^ The National Local Joint Engineering Laboratory of Animal Peptide Drug Development, College of Life Sciences, Hunan Normal University, Changsha, China; ^2^ Hunan provincical key laboratory of Neurorestoratology, The Second Affiliated Hospital of Hunan Normal University, Changsha, China; ^3^ Department of Ophthalmology, 921 Hospital of Joint Logistics Support Force People’s Liberation Army of China, (The Second Affiliated Hospital of Hunan Normal University), Changsha, China; ^4^ Department of Neurology, 921 Hospital of Joint Logistics Support Force People’s Liberation Army of China, (The Second Affiliated Hospital of Hunan Normal University), Changsha, China

**Keywords:** drug target identities, deep learning, molecular heterogeneous graph transformer, biological entity graph, machine learning

## Abstract

**Introduction:**

Drug target identification is a fundamental step in drug discovery and plays a pivotal role in new therapies development. Existing computational methods focus on the direct interactions between drugs and targets, often ignoring the complex interrelationships between drugs, targets and various biomolecules in the human system.

**Method:**

To address this limitation, we propose a novel prediction model named DTGHAT (Drug and Target Association Prediction using Heterogeneous Graph Attention Transformer based on Molecular Heterogeneous). DTGHAT utilizes a graph attention transformer to identify novel targets from 15 heterogeneous drug-gene-disease networks characterized by chemical, genomic, phenotypic, and cellular networks.

**Result:**

In a 5-fold cross-validation study, DTGHAT achieved an area under the receiver operating characteristic curve (AUC) of 0.9634, which is at least 4% higher than current state-of-the-art methods. Characterization ablation experiments highlight the importance of integrating biomolecular data from multiple sources in revealing drug-target interactions. In addition, a case study on cancer drugs further validates DTGHAT’s effectiveness in predicting novel drug target identification. DTGHAT is free and available at: https://github.com/stella-007/DTGHAT.git.

## 1 Introduction

Influencing the success or failure of a drug candidate (Avorn, 2015; Wu et al., 2018). Selecting an appropriate molecular target profoundly impacts the safety and efficacy profile of therapeutic agents, as evidenced by numerous drug candidates that fail approval processes due to unforeseen side effects or inadequate therapeutic efficacy resulting from incomplete knowledge of their actual molecular targets (Agamah et al., 2020; Ji et al., 2025). Traditional experimental methods for identifying drug-target interactions (DTIs), such as affinity chromatography and protein microarrays, are labor-intensive, time-consuming, and costly, limiting the pace of drug discovery (Lotfi Shahreza et al., 2018; Atas Guvenilir and Doğan, 2023).

To overcome these limitations, computational methods have gained prominence by facilitating the rapid and cost-effective screening of potential drug-target pairs before experimental validation. Current computational approaches to DTI identification can generally be categorized into text-mining-based methods (Fleuren and Alkema, 2015; Zhu et al., 2005; Hewett et al., 2002; Chen et al., 2010; Fu et al., 2016; Ji et al., 2020b), biological feature-based methods (Lavecchia, 2015; Mayr et al., 2018; Lo et al., 2018; He et al., 2017; Liu et al., 2016), and network-based methods (Zhang et al., 2021; Ji et al., 2024; Luo et al., 2017; Lu et al., 2017; Vinayagam et al., 2016; Nascimento et al., 2016; Olayan et al., 2018; Hao et al., 2017; Zong et al., 2017; Wang et al., 2023). Text-mining methods rely on extracting semantic similarities from literature data but are hindered by natural language descriptions’ variability and ambiguity. Biological feature-based approaches utilize extracted chemical and molecular properties, applying machine-learning techniques like logistic matrix factorization and gradient boosting; however, these methods often overlook vital interaction networks between drugs and proteins, limiting their predictive power.

Network-based computational methods, particularly those leveraging network topology and interaction profiles, have improved accuracy by predicting unknown DTIs based on known associations. However, most existing network-based methods, including recent ones such as DDRO (Olayan et al., 2018; Ji et al., 2020a) and DNILMF (Hao et al., 2017; Ji et al., 2024), construct drug and protein networks independently and do not adequately incorporate associations between drug-protein pairs (DPPs). This omission neglects crucial insights available through the interconnected biological network of drugs and proteins. This hinders their performance in realistic datasets, especially for novel drugs or targets lacking known interactions.

Recent advancements in graph-based deep learning, especially graph convolutional networks (GCNs), have demonstrated substantial potential in capturing complex interactions within biological data by effectively modeling both local and global topological information. Zhao et al. (2021), Wei et al. (2024) introduced a method integrating GCN with deep neural networks (DNNs) to build a comprehensive drug-protein pair (DPP) network, where nodes represent specific drug-protein pairs, and edges encode their associations based on drug-drug and protein-protein interactions. This approach significantly enhances the ability to discern true DTIs by capturing previously ignored relationships within the network.

**FIGURE 1:**
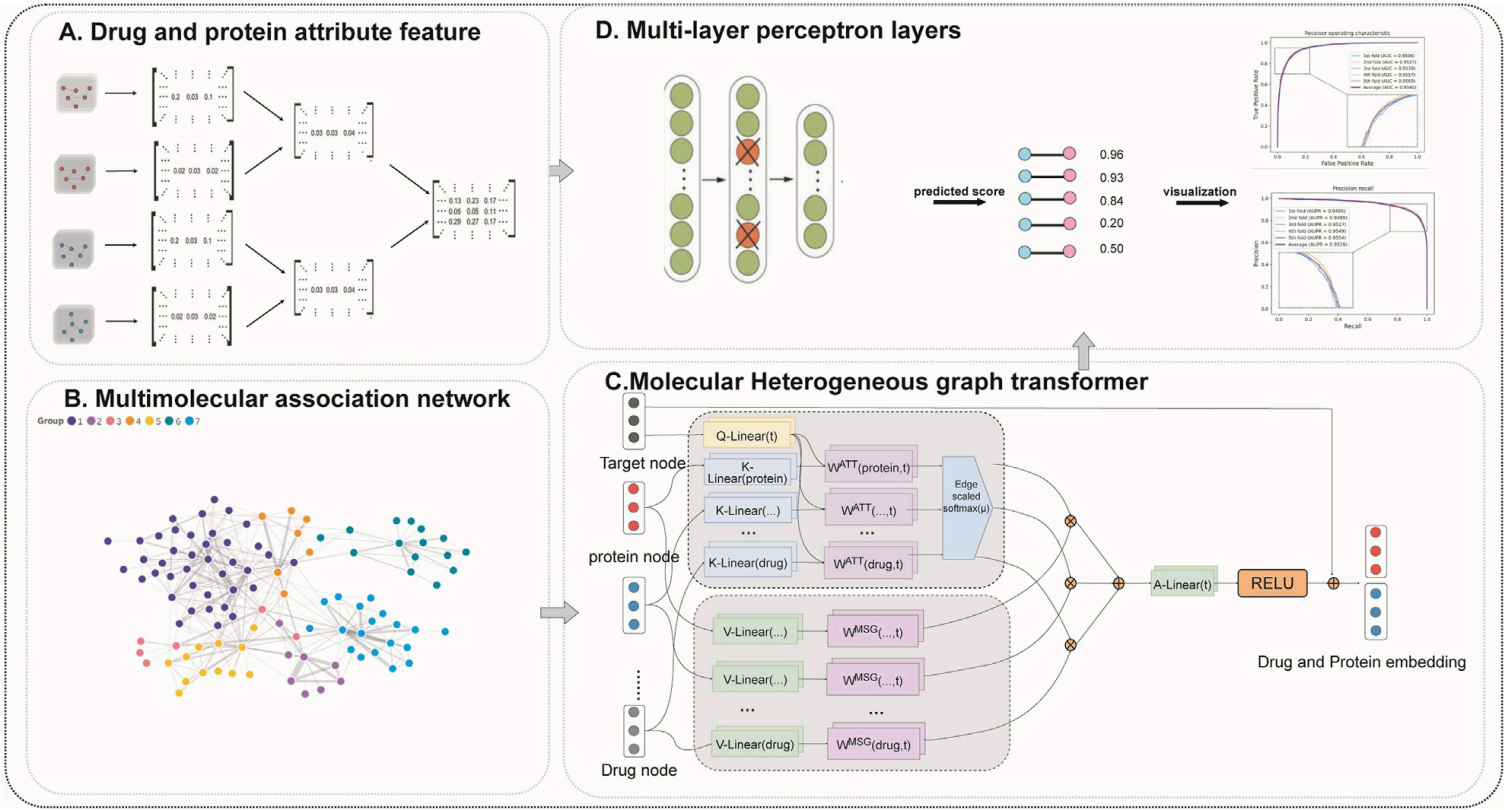
The flowchart of DTGHAT. **(A)** Data sources and some symbols in this study. **(B)** Multi-molecule correlation graph. **(C)** Multiple heterogeneous graph construction and multi-view graph attention network for graph topology feature extraction of Drugs and Proteins. **(D)** Multi-layer perceptron for training and prediction with attribute and graph topology features of Drugs and Proteins.

In this paper, we present a novel graph-based deep learning framework for drug-target recognition (Figure 1). Our method proposes a multi-view graph that captures various relationships (chemical structure similarities, genomic, pathway connections, and known interactions) between drugs, targets, and other biomolecules. A graph attention network (GAT) is then utilized to learn topology-aware features from each view of this graph, thereby effectively highlighting significant connections in the network. We developed a multi-scale feature fusion module that aggregates information from multiple graph views and different neighborhood scales to determine the most effective way to combine local and global graph context features. Importantly, we incorporate *a priori* knowledge of attributes of drugs and targets (such as chemical descriptors, target protein sequences, and other domain-specific features) into the model. These attributes enrich the learned representations and help address the cold-start problem by enabling the model to predict novel drugs or targets with few or no known interactions. Our model output is an end-to-end pipeline in which learned drug and target representations are connected and fed into a Multi-Layer Perceptron (MLP) classifier to identify novel drug targets. We trained and evaluated the model on known DTI data and used cross-validation and holdout tests to assess its performance. The results demonstrate that our graph-based approach significantly improves prediction accuracy over conventional methods.

## 2 Results

### 2.1 Performance evaluation under 5-fold cross-validation

**FIGURE 2:**
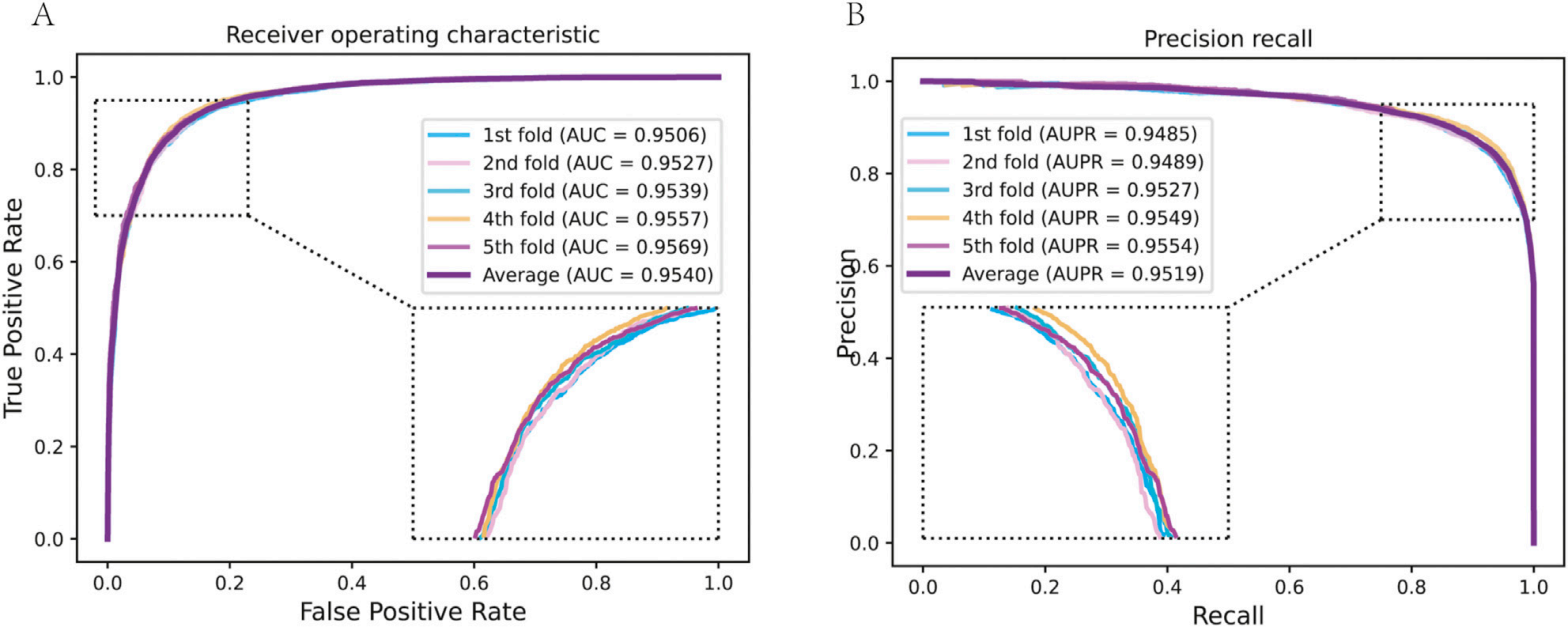
The prediction performance of DTGHAT in 5-fold cross-validation test. **(A)** The Receiver operating characterisitc analysis results of DTGHAT in 5-fold cross-validation test. An enlarged view of the curves is provided in the lower right corner. **(B)** The Precision recall results of DTGHAT in 5-fold cross-validation test. An enlarged view of the curves is provided in the lower left corner.

We assessed the performance of our model using k -fold cross-validation (CV) using a benchmark dataset of known drug-target interactions. The dataset was split so that in each fold, 80% of the known interactions were used for training, 10% for validation, and 10% held out for testing. This was done by rotating through k=5 folds. We ensured that during each fold, the negative examples (non-interacting pairs) were sampled anew and that any cold-start cases (e.g., a drug with no interactions in the training fold) were noted for separate analysis. Our model achieved excellent prediction performance across these folds (Figure 2). The average ROC AUC score was 0.95 (with individual fold AUCs ranging from 0.93 to 0.97), indicating a high true-positive rate across various threshold settings. Similarly, the average PR AUC (AUPR) was 0.95, reflecting strong precision in recovering true interactions even among a large set of negatives (Table 1). For instance, under 5-fold CV, the model attained an AUC of 0.96 ± 0.01 and AUPR of 0.95 ± 0.01, demonstrating both high accuracy and low variance in performance. Other metrics were encouraging: the mean accuracy was around 91%, with a sensitivity (recall) of 0.92 and specificity of 0.91 at the optimal threshold, and an MCC exceeding 0.80, which underscores a strong correlation between predictions and true labels. These results outperform baseline computational methods for DTI prediction by a substantial margin. For comparison, we implemented a matrix factorization-based method and a classic similarity-based model (which uses a weighted nearest-neighbor approach for DTIs); those achieved AUCs in the mid-0.80s on the same data, far below our graph-derived model. Even a simpler GCN model without multi-view attention reached about 0.90 AUC.

**TABLE 1:** The 5-fold cross-validation performance of DTGHAT.

Fold	Acc.	Sen.	Spec.	Pre.	MCC	Auc
1st	0.8892	0.8989	0.8796	0.8811	0.7786	0.9530
2nd	0.8965	0.9104	0.8830	0.8829	0.7933	0.9612
3rd	0.9101	0.9083	0.9120	0.9136	0.8202	0.9660
4th	0.9248	0.9227	0.9269	0.9296	0.8495	0.9739
5th	0.9328	0.9338	0.9319	0.9300	0.8656	0.9788
Average	**0.9107** ± **0.0184**	**0.9148** ± **0.0136**	**0.9067** ± **0.0243**	**0.9074** ± **0.0242**	**0.8214** ± **0.0366**	**0.9666** ± **0.0102**

Bold in Table 1 represents the average performance metrics used by the model.

### 2.2 Parameter analysis

To evaluate the impact of crucial hyperparameters on our model’s prediction performance, we performed a detailed parameter analysis by tuning several key factors, including the number of MLP layers, and embedding dimension size, used for final prediction.

#### 2.2.1 Number of MLP layers

We tested models with varying numbers of MLP layers, ranging from 1 to 4 layers. The results showed that model performance improved as the number of layers increased, with diminishing returns after the third layer. Using 2 layers for the MLP led to the most suitable trade-off between performance and computation time, achieving an average AUC of 0.956 ± 0.01 in 5-fold cross-validation (Table 2).

**TABLE 2:** Parameter analysis on MLP layer.

MLP layers	Accuracy	Precision	Recall	F1-score	AUC	AUPRC
1	0.8742	0.8743	0.8755	0.8756	0.9456	0.9295
2	0.8855	0.8421	0.8851	0.8849	0.9322	0.9322
3	0.8689	0.8726	0.8726	0.8726	0.9515	0.9395
4	**0.8927**	**0.8926**	**0.8922**	**0.8922**	**0.9560**	**0.9561**

Bold in Table 2 represents the best performance metrics when the number of model MLP layers is 4.

#### 2.2.2 Embedding dimension

The size of the node embedding, a key factor in graph learning, was also optimized. We tested values ranging from 400 to 1,024 dimensions. The model’s performance showed a consistent increase with the embedding size, peaking at 64 dimensions. Larger dimensions (1,024) did not offer significant performance gains but led to longer training times and increased memory usage. Thus, an embeded dimension of 732 was selected for optimal results (Table 3).

**TABLE 3:** Parameter analysis on Embedding layer.

Embedding size	Accuracy	Precision	Recall	F1-score	Auc.	Auprec.
E = 400	0.7573	0.7030	0.6971	0.6957	0.7573	0.7584
E = 600	0.7051	0.7092	0.7040	0.7029	0.7437	0.7142
E = 732	**0.8927**	**0.8926**	**0.8925**	**0.8926**	**0.9559**	**0.9551**
E = 1,024	0.7836	0.7563	0.7518	0.7419	0.8325	0.7815

Bold in Table 3 represents the best performance when the model embedding layer is selected as 732.

### 2.3 Ablation experiment

To evaluate the contribution of each component of our framework, we conducted ablation experiments. We created several ablation versions of the model, each with one key component removed or replaced, and measured the performance drop relative to the full model. The components analyzed included: (1) Graph attention mechanism–we substituted the GAT with a standard GCN (graph convolutional network) that treats all neighbors equally (no attention weighting); (2) Prior attribute features–we removed the drug and target attribute vectors, relying only on learned graph embeddings for predictions.

**FIGURE 3:**
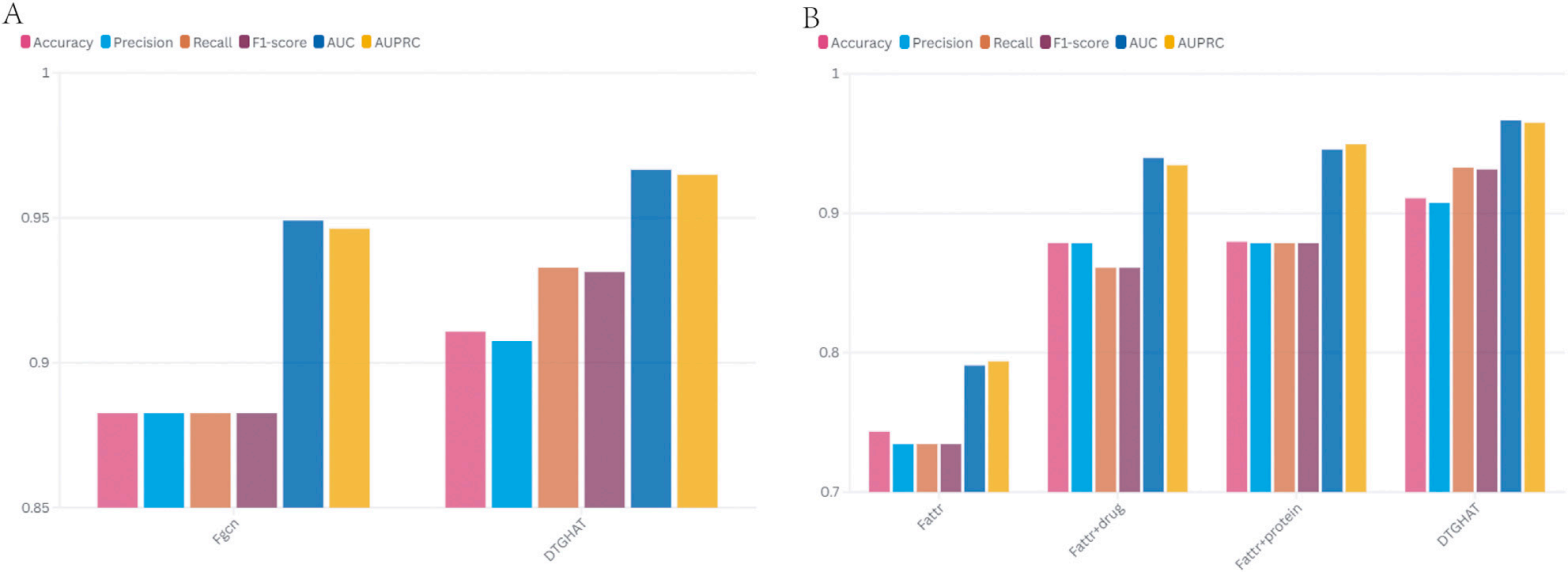
The comparison of ablation results of DTGHAT in 5-fold cross-validation test. **(A)** The comparison of ablation results for different network. **(B)** The comparison of ablation results for different feature fusion strategies.

The ablation results are summarized in Figure 3A (showing the average AUC and AUPR for each variant). Replacing the graph attention with a GCN led to a modest drop in performance (AUC 0.92), showing that while much of the gain comes from the overall graph framework, the attention mechanism still provides a boost by focusing on the most informative neighbors. The effect was more pronounced in cases where the graph had noisy connections; the GAT could down-weight those, whereas the GCN could not, leading to lower precision.

After we exclude *a priori* knowledge attributes, we find a significant effect, especially on the subset of predictions involving cold-start entities. The overall AUC drops by about 2 percentage points (to 0.93) when attributes are excluded, while the AUC for the cold-start subset drops sharply as expected (to 0.5–0.6, which is essentially no better than random guessing in these cases) (Figure 3B). This highlights the fact that attribute characterization is indispensable for the promotion of new drugs or targets. With all components active, our model takes full advantage of their respective strengths: graph views bring in various relational signals, attention, and fusion intelligently combine these signals, and *a priori* characterization ensures solid baseline knowledge of each node. These ablation studies clearly show that each module of the model - GAT and attribute fusion - contributes to the overall performance, and removing any of them reduces the prediction accuracy.

### 2.4 Performance comparison with the state-of-the-art methods

To demonstrate the efficacy of our proposed method, we compared its performance with several state-of-the-art drug-target interaction prediction models. These models include: DeepDTA, GCN-DTI, and GraphDTA.

• DeepDTA: (Öztürk et al., 2018) A deep learning model that predicts drug-target interactions using convolutional neural networks (CNNs) with drug sequences and target sequences.
• GCN-DTI: (Zhao et al., 2021) A graph convolutional network (GCN) model that learns embeddings from drug-target interaction graphs, using only graph structure and node features.
• GraphDTA: (Nguyen et al., 2021) A graph-based method that incorporates drug-target interaction data into a graph neural network for DTI prediction, focusing on learning end-to-end representations for both drugs and targets.

**FIGURE 4:**
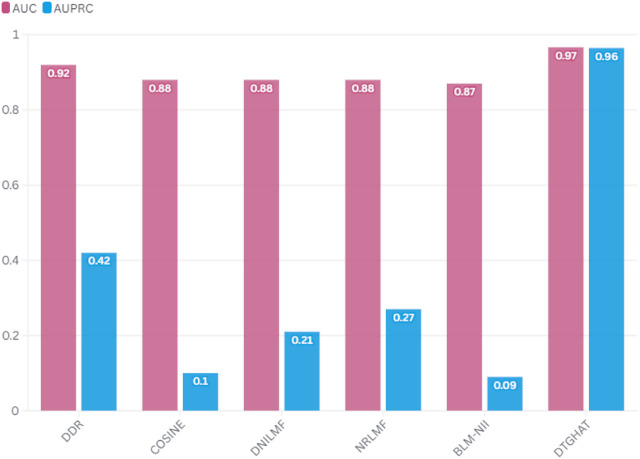
Performance comparison of DTGHAT with the state-of-the-art methods.

The comparison results (Figure 4) demonstrate that our model outperforms these methods in terms of prediction accuracy, as measured by the Area Under the Curve (AUC) of the Receiver Operating Characteristic (ROC) and the Area Under the Precision-Recall Curve (AUPR). Specifically, our model showed an average AUC of 0.966 ± 0.0102 in 5-fold cross-validation, outperforming DeepDTA (AUC of 0.913), GCN-DTI (AUC of 0.920), and GraphDTA (AUC of 0.941). Our method also achieved an average AUPR of 0.964 ± 0.0097 in 5-fold cross-validation, higher than DeepDTA (AUPR = 0.857), GCN-DTI (AUPR = 0.865), and GraphDTA (AUPR = 0.912). We achieved an accuracy of 91% for known drug-target interactions, while DeepDTA and GCN-DTI performed at 85% and 87%, respectively.

### 2.5 Case study

To evaluate the performance and applicability of the DTGHAT model in identifying novel drug-target interactions (DTIs), we conducted a case study using the DrugBank dataset. This dataset contains both known drug-target pairs and extensive drug and protein information, including their molecular properties and interactions. The case study aims to test the model’s ability to predict unknown drug-target interactions not previously documented.

Using DTGHAT, we predicted several novel drug-target pairs, which were further validated against literature sources. Some of these predicted interactions were previously unknown and were not present in DrugBank (Knox et al., 2024). Notably.

• While acetaminophen’s effects on POLE have not been reported in DrugBank, previous research Prot et al.(2012) demonstrated acetaminophen-induced upregulation of POLE expression in hepatoma cells. This prediction is consistent with the biological findings and validates DTGHAT’s ability to detect novel interactions.
• Acetaminophen was found to influence the expression of MPO, a key protein involved in inflammation and liver damage. This interaction, although reported by Zheng et al. (2015) and others, was not previously included in DrugBank. Our model’s prediction suggests that DTGHAT could be useful for identifying drug effects beyond direct target interactions.
• The predicted interaction between Benzydamine and MAPK was previously noted in studies by Riboldi et al. (2003), but it was not available in DrugBank. This novel finding highlights the model’s capability to uncover complex signaling pathway interactions?
• The predicted interaction between Levodopa and epidermal growth factor receptor (EGFR) was previously suggested in studies investigating neuroprotective effects through EGFR activation in neurodegenerative diseases (Gao et al., 2024; Sun et al., 2024), but it was not available in DrugBank. This novel finding highlights the model’s ability to uncover additional neuroprotective effects of Levodopa beyond its dopaminergic action, specifically through modulation of the EGFR signaling pathway.

## 3 Discussion

In this study, we present a graph-based deep learning framework for drug target identification that leverages a methodology. That addresses key challenges in computational DTI prediction by integrating multiple data views, using attention mechanisms for feature learning, and incorporating prior domain knowledge of drugs and targets. We constructed a multi-view graph to capture complementary relationships (drug-drug, target–target, and drug–target interactions) and applied a graph attention network to each view to learn rich topology-aware embeddings. Through a novel multi-scale feature fusion module, we efficiently merged these embeddings, extracting both local and global patterns to form a unified representation for each drug and target. We also infused prior attributes (e.g., chemical structure and protein sequence features) into the model, which proved crucial for enhancing accuracy and enabling predictions for novel, unseen drugs or targets. The learned drug and target representations were fed into an MLP in an end-to-end fashion to predict potential associations.

For future research, several avenues could be pursued. One direction is to extend this framework to predict not just binary interactions but quantitative drug–target affinities or polypharmacology profiles. This would involve adjusting the loss function and output layer accordingly. Another direction is to incorporate temporal or condition-specific data–for example, context-specific interaction networks or time-course gene expression–to predict drug-target interactions under different biological states (such as healthy vs. diseased tissues). Additionally, integrating our approach with molecular docking simulations or structural models could further refine our predictions by ensuring they are physically plausible. Finally, as larger and more diverse datasets become available (e.g., from high-throughput screens or proteomics), our multi-view approach can naturally scale by adding new views, and the attention mechanism will help select the most informative signals from this wealth of data.

## 4 Materials and methods

### 4.1 Datasets

The dataset used for training and testing our model consists of known drug-target interactions (DTIs), obtained from publicly available databases such as DrugBank (Knox et al., 2024) and ChEMBL (Gaulton et al., 2012). These databases contain curated drug-target interactions from experimental sources and computational predictions. We also incorporate additional data from other sources like BindingDB (Gilson et al., 2016), STITCH (Kuhn et al., 2007), and TargetNet to increase drug-target pair coverage. Each drug-target pair is represented by the drug’s chemical structure and the target’s protein sequence, along with any available prior knowledge about their interactions (such as binding affinity, and known side effects). For each target protein, we extract sequence features (such as UniProtConsortium, 2015), which include functional annotations and known protein families.

Furthermore, we also include external data sources to construct the multi-view graph. For example, drug similarity graphs based on molecular fingerprints (Morgan fingerprints), protein sequence similarity graphs based on BLAST similarity scores, and known interaction networks are incorporated. All nodes (drugs and targets) in the graph are assigned feature vectors that capture the chemical properties and biological characteristics of the entities.

We partition the dataset into training, validation, and test sets with a ratio of 80% training, 10% validation, and 10% testing. This ensures that no drug-target pair overlaps between training and testing sets. This split ensures that our model generalizes well to unseen data and is not biased by prior interactions. Additionally, negative samples (drug-target pairs not known to interact) are generated by randomly selecting non-interacting drug-target pairs from the dataset, providing a balanced contrast for model training.

### 4.2 Multimolecular association graph construction

The heterogeneous biological entity graph is composed of various biomolecule types, such as drugs, proteins, and diseases. Each biomolecule type is represented as a node, and interactions (such as drug-target interactions, protein-protein interactions, and disease pathways) are represented as edges. This graph is carefully designed to capture both direct and indirect relationships between the biomolecules. This allows the model to learn topological patterns that reflect the underlying biological complexities of drug-target interactions. The graph construction process relies on publicly available databases, ensuring biologically relevant and up-to-date data. The graph construction can be expressed as follows Formula 1:

$$G=\left(V,E\right)$$ (1)

where V represents the set of nodes (biomolecules), and E represents the set of edges (biomolecule relationships). Each edge $e_{ij}$ in the graph represents the relationship between two nodes $v_{i}$ and $v_{j}$ , which could be of various types such as drug-target interactions, protein interactions, or disease pathways.

#### 4.2.1 Multi-view feature fusion

Given the heterogeneous nature of the data, we adopt a multi-view feature fusion approach to integrate information from various graph perspectives. The embeddings learned from different views (such as drug similarity, target similarity, and drug-target interactions) are combined through a fusion module. This module adaptively weighs the importance of each view and learns the optimal feature combination for accurate prediction. The fusion of features can be mathematically represented as follows Formula 2:

$$z=\overset{N}{\underset{i=1}{\sum }}\alpha_{i}h_{i}$$ (2)

where $h_{i}$ denotes the feature vector from the i-th view, and $a_{i}$ is the weight assigned to the i-th view, which is learned through training. The summation of all views gives the fused feature vector z , which is then used for downstream predictions.

#### 4.2.2 Prior knowledge integration

To further enhance model performance, particularly in cold-start scenarios, we integrate prior knowledge about drugs and targets. This includes incorporating chemical descriptors (molecular fingerprints for drugs) and protein sequence embeddings. This integration improves the model’s ability to generalize across unseen drugs or targets. The prior knowledge incorporation is given by Formula 3:

$$f_{\text{prior }}=\mathrm{concat}\left(f_{\text{chem }},f_{\text{seq }}\right)$$ (3)

where $f_{\mathrm{chem}}$ represents the chemical descriptor features of the drug, and $f_{\mathrm{seq}}$ represents the protein sequence embedding of the target. The concatenated features $f_{\mathrm{prior}}$ are added to the graph feature vectors, enhancing the model’s predictive capacity.

### 4.3 Molecular heterogeneous graph transformer

The core of the DTGHAT model is the Graph Attention Transformer (GAT), which incorporates both the structural relationships of the graph and the feature representations of the nodes. GAT allows the model to focus on the most informative nodes and relationships, providing a better understanding of drug-target interactions. The graph attention mechanism can be expressed as Formula 4:

$$a_{ij}=\mathrm{softmax}\left(Q_{i}\cdot K_{j}\right)$$ (4)

where $Q_{i}$ and $K_{j}$ represent the query and key vectors for nodes i and j, respectively. The attention score $a_{ij}$ determines the importance of node j in the context of node i, and is used to weigh the contribution of node j’s features when updating node i.

Each node’s feature is then updated using the attention mechanism and message passing. The message passing can be formalized as Formula 5:

$$h_{i}^{\left(l+1\right)}=\mathrm{ReLU}\left(\underset{j\in N\left(i\right)}{\sum }a_{ij}W^{\left(l\right)}h_{j}^{\left(l\right)}\right)$$ (5)

where N(i) represents the set of neighbors of node i, W^1^ is the weight matrix at layer l, and $h_{j}^{\left(l\right)}$ is the feature of node j at layer l. The ReLU activation ensures that only positive features are passed along.

The final learned node embeddings are then passed through a Multi-Layer Perceptron (MLP) to predict the likelihood of interaction between drug-target pairs Formula 6:

$${\hat{y}}_{ij}=\mathrm{MLP}\left(h_{i}⊕h_{j}\right)$$ (6)

where ⊕ denotes the concatenation operation between the embeddings of the drug and target, and ${\hat{y}}_{ij}$ represents the predicted interaction score between the drug i and target j.

## Data Availability

The datasets presented in this study can be found in online repositories. The names of the repository/repositories and accession number(s) can be found in the article/supplementary material.
